# Dual role of interleukin-17 in pannus growth and osteoclastogenesis in rheumatoid arthritis

**DOI:** 10.1186/ar3238

**Published:** 2011-02-04

**Authors:** Hiroshi Ito, Hidehiro Yamada, Toshiko N Shibata, Hirofumi Mitomi, So Nomoto, Shoichi Ozaki

**Affiliations:** 1 Division of Rheumatology and Allergology, Department of Internal Medicine, St. Marianna University School of Medicine, 2-16-1 Sugao, Miyamae-ku, Kawasaki 216-8511, Japan; 2 Department of Orthopaedic Surgery and Rheumatology, Saiseikai Yokohamashi Tobu Hospital, 3-6-1, Shimosueyoshi, Tsurumi-ku, Yokohama 230-8765, Japan

## Abstract

**Introduction:**

In a murine model, interleukin (IL)-17 plays a critical role in the pathogenesis of arthritis. There are controversies, however, regarding whether IL-17 is a proinflammatory mediator in rheumatoid arthritis (RA). We previously established an *ex vivo *cellular model using synovial tissue (ST)-derived inflammatory cells, which reproduced pannus-like tissue growth and osteoclastic activity *in vitro*. Using this model, we investigated the effects of IL-17 on pannus growth and osteoclastogenesis in RA.

**Methods:**

Inflammatory cells that infiltrated synovial tissue from patients with RA were collected without enzyme digestion and designated as ST-derived inflammatory cells. ST-derived inflammatory cells were cultured in the presence or absence of IL-17 or indomethacin, and the morphologic changes were observed for 4 weeks. Cytokines produced in the culture supernatants were measured by using enzyme-linked immunosorbent assay kits. Osteoclastic activity was assessed by the development of resorption pits in calcium phosphate-coated slides.

**Results:**

Exogenous addition of IL-17 dramatically enhanced the spontaneous production of IL-6 and prostaglandin E_2 _(PGE_2_) by the ST-derived inflammatory cells, while it had no effect on the production of tumor necrosis factor (TNF)-α and macrophage colony-stimulating factor (M-CSF). Furthermore, IL-17 did not affect the spontaneous development of pannus-like tissue growth and osteoclastic activity by the ST-derived inflammatory cells. On the other hand, IL-17 enhanced pannus-like tissue growth, the production of TNF-α and M-CSF and the development of osteoclastic activity in the presence of indomethacin, an inhibitor of endogenous prostanoid production, while exogenous addition of PGE_1 _suppressed their activities.

**Conclusions:**

The present study suggests that IL-17 induces negative feedback regulation through the induction of PGE_2_, while it stimulates proinflammatory pathways such as inflammatory cytokine production, pannus growth and osteoclastogenesis in RA.

## Introduction

Rheumatoid arthritis (RA) is chronic autoimmune inflammatory disease that ultimately leads to the progressive destruction of cartilage and bone in numerous joints. Proinflammatory cytokines such as tumor necrosis factor (TNF)-α [1], interleukin (IL)-1 [2] and IL-6 [3] were produced from synovial tissue (ST), which maintains its inflammatory condition. Inflammation of synovial membrane results in the development of aggressive granulation tissue, called pannus. Pannus tissue is composed mainly of inflammatory cells such as macrophages and fibroblast-like synoviocytes (FLSs) [4].

At present, TNF-α and IL-6 are among the most important targets of therapy, and blocking TNF-α results in a rapid and sustained improvement of clinical signs and symptoms [5-7]. Anti-TNF therapy also prevents radiological progression of joint destruction [8-10]. Anti-IL-6 receptor monoclonal antibody (mAb) (tocilizumab) has also proved to reduce disease activity, even in patients who had an insufficient response to anti-TNF therapy, and to inhibit the progression of structural joint damage [11-13]. These clinical experiences suggest that there are at least two pathways, TNF-α-dependent and IL-6-dependent, leading to the progression of pannus growth and joint destruction in RA.

Recent studies have demonstrated critical roles of IL-17, which is produced by a newly identified subset of CD4^+ ^T cells, Th-17, in animal models of arthritis [14,15]. In humans, IL-17 is a potent inducer of other proinflammatory cytokines, such as TNF-α, IL-1β, IL-6 and IL-8 from monocytes and/or macrophages or synovial fibroblasts [16,17]. IL-17 has been detected in synovial fluids of RA [18,19]. These findings suggest that IL-17 is an important cytokine located upstream of the two pathways, TNF-α-dependent and IL-6-dependent. Preliminary clinical trial using humanized anti-IL-17 mAb has shown an improvement of clinical signs and symptoms of RA [20]. It is still unknown, however, whether inhibition of IL-17 prevents joint destruction in RA.

To further confirm the hypothesis, the present study was undertaken to clarify a role of IL-17 in RA using our recently established *ex vivo *human cellular model, where rheumatoid ST-derived inflammatory cells spontaneously develop pannus-like tissue *in vitro *and osteoclastic bone resorption [21].

## Materials and methods

### Reagents

IL-17 was purchased from PeproTech (Rocky Hill, NJ, USA). PGE_1 _was purchased from Sigma-Aldrich (St. Louis, MO, USA). Indomethacin was obtained from Wako (Osaka, Japan).

### Synovial tissue specimens

ST specimens were obtained from patients who fulfilled the American College of Rheumatology criteria for RA who underwent knee joint replacement. In compliance with institutional policies, informed consent was obtained from all patients. The study was approved by the ethics committee of each institution.

### *In vitro* reconstruction of inflammatory tissue by ST-derived inflammatory cells

ST-derived inflammatory cells were prepared as previously described [21]. In brief, ST specimens were cut into small pieces and cultured in 100-mm dishes containing RPMI-1640 (Asahi Technoglass, Chiba, Japan) with 10% fetal calf serum (FCS) and 1,000 U/ml penicillin G sodium-streptomycin sulfate (Gibco BRL, Grand Island, NY, USA). After 1 to 3 days' incubation, tissue was removed and single cells were collected by vigorous pipetting. Cell suspensions were washed once, and viable cells were collected into Lymphocyte Separation Medium (Nacalai Tesque, Kyoto, Japan). Single suspensions of ST-derived inflammatory cells were seeded at a density of 5 × 10^5^/well in 48-well culture plates and cultured in Dulbecco's modified Eagle's medium (DMEM; Gibco BRL) containing 10% FCS, 100 U/ml penicillin G sodium and 100 μg/ml streptomycin sulfate. The culture was observed for morphologic changes under an inverted phase-contrast microscope twice a week for 4 weeks. When cultured in DMEM and 10% FCS in the absence or presence of IL-17 (0.1 to 100 ng/ml) or indomethacin (100 nM to 1 μM), ST-derived inflammatory cells started to aggregate, forming foci within a few days. Further culturing resulted in three-dimensional (3-D) growth, which ultimately produced macroscopic tissue 2 mm in size within 4 weeks. Morphologic changes were semiquantitatively scored on a scale of 0 to 4, according to the degree of tissue development, where 0 was no cellular foci or aggregations, 1 was the formation of cellular foci or aggregation, 2 was further growth of cellular aggregations, 3 was further 3-D growth with a multilayered structure, and 4 was the development of macroscopic tissue. Cumulative tissue growth score was calculated by the total sum of the tissue growth scores obtained twice weekly for 4 weeks of culture. Half of the supernatants were collected twice weekly and replaced with fresh medium or the addition of a half dose of IL-17 or indomethacin. Supernatants were frozen at -80°C until assayed.

### Cytokine assay

ST-derived inflammatory cells were seeded in 48-well culture plates (5 × 10^5^/well) and cultured in DMEM and 10% FCS.

Half of the supernatants were collected three times per week and replaced with fresh medium. Supernatants were frozen at -80°C until assayed, and levels of IL-6, PGE_2_, TNF-α and M-CSF (all from R&D Systems, Minneapolis, MN, USA) released into the culture supernatants were measured using enzyme-linked immunosorbent assay kits according to the manufacturers' recommendations.

### Bone resorption assay

ST-derived inflammatory cells were seeded (1 × 10^5 ^cells/well) onto calcium phosphate-coated slides (Osteologic; BD Biosciences, MA, USA) and incubated in RPMI-1640 with 1% FCS, 50 μg/ml ascorbic acid (Sigma) and 10 mM β-glycerophosphate (Sigma) for 7 to 14 days in a CO_2 _incubator (5% CO_2_, 100% humidity at 37°C). Half of the supernatants were replaced with fresh medium once weekly. The calcium phosphate-coated slides were washed with distilled water and bleach solution (6% NaOCl and 5.2% NaCl) and then air-dried. The number of resorption pits were counted under a microscope.

## Results

### IL-17 enhances IL-6 and PGE_2 _production by ST-derived inflammatory cells

**Figure 1:**
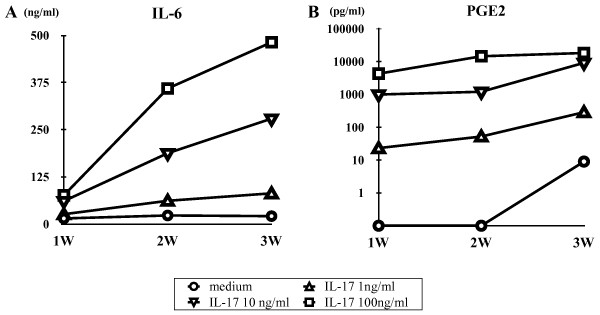
**Effect of interleukin (IL)-17 on the production of (A) IL-6 (A) and (B) prostaglandin E_2 _(PGE_2_) by the synovial tissue (ST)-derived inflammatory cells**. Cells were incubated in the absence or presence of increasing concentrations of IL-17 (0 to 100 ng/ml) for 3 weeks. IL-6 (*n *= 7) and PGE_2 _(*n *= 3) in the culture supernatants were measured by enzyme-linked immunosorbent assay as described in Materials and methods.

Using a recently established *ex vivo *cellular model of RA, we examined the effect of IL-17 on the production of IL-6 and PGE_2 _by the ST-derived inflammatory cells. During the cell culture, ST-derived inflammatory cells spontaneously produced IL-6 and PGE_2 _in the supernatant as shown in Figure 1. Addition of IL-17 into the culture resulted in the enhancement of both IL-6 and PGE_2 _production in a dose-dependent manner.

### Effect of IL-17 on the development of pannus-like inflammatory tissue *in vitro* by the ST-derived inflammatory cells

**Figure 2:**
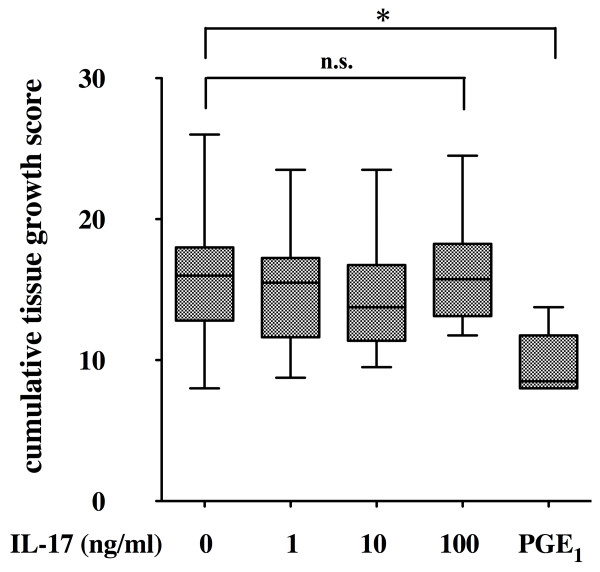
**Effect of interleukin (IL)-17 and prostaglandin E_1 _(PGE_1_) on pannus-like tissue growth *in vitro***. Synovial tissue (ST)-derived inflammatory cells were incubated in the absence or presence of increasing concentrations of IL-17 (0 to 100 ng/ml) (*n *= 17) or PGE_1 _(100 nM) (*n *= 9). Morphologic changes were observed under an inverted phase contrast microscope twice weekly for 4 weeks and were scored semiquantitatively on a scale of 0 to 4 according to the degree of tissue development as described in Materials and methods. Box and whisker plots indicate 25th/75th percentile and minimum/maximum, respectively. Bar indicates the median value. *P < 0.001 (by Mann-Whitney *U *test).

We have reported that ST-derived inflammatory cells showed spontaneous development of pannus-like tissue *in vitro *[21]. The ST-derived inflammatory cells at the beginning of the culture contained 1.6% to 4.2% FLSs (mean, 2.6%), 35.8% to 65.7% macrophages (mean, 53.7%) and 32.4% to 62.6% small lymphocytes (mean, 44.7%) when assessed by morphological observation. During the culture of ST-derived inflammatory cells, marked proliferation and migration of the FLSs into the pannus-like tissue were observed. At the end of culture, pannus-like tissue contained more than 80% FLSs and less than 10% of macrophages and T cells as assessed by immunohistochemistry. As IL-17 enhanced IL-6 and PGE_2 _production by the ST-derived inflammatory cells, we investigated the effect of IL-17 on the development of pannus-like tissue *in vitro*. The cumulative tissue growth score during 4 weeks of culturing of ST-derived inflammatory cells was not affected by the addition of IL-17 up to 100 ng/ml, while it was suppressed by the exogenous addition of 100 nM PGE_1 _(Figure 2) as well as 100 nM PGE_2 _(data not shown).

**Figure 3:**
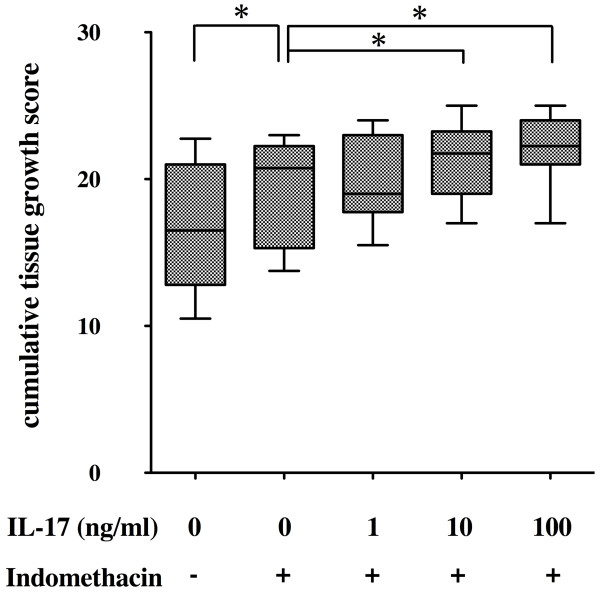
**Effect of interleukin (IL)-17 on pannus-like tissue growth in the presence of indomethacin**. Synovial tissue (ST)-derived inflammatory cells (*n *= 7) were incubated with an incremental dose of IL-17 in the absence or presence of indomethacin (100 to 1000 nM). Morphologic changes were observed under an inverted phase contrast microscope twice weekly for 4 weeks and were scored semiquantitatively on a scale of 0 to 4 according to the degree of tissue development as described in Materials and methods. Box and whisker plots indicate 25th/75th percentile and minimum/maximum, respectively. Bar indicates the median value. *P < 0.05 (by Wilcoxon signed-rank test).

These results suggested that the effect of IL-17 on the development of pannus-like tissue was modified by IL-17-enhanced endogenous PGE_2 _production. To confirm this possibility, we investigated the effect of indomethacin, an inhibitor of endogenous prostanoids, on the pannus-like tissue growth *in vitro*. Addition of indomethacin resulted in a significant enhancement of the *in vitro *tissue growth by the ST-derived inflammatory cells (Figure 3). In the presence of indomethacin, the *in vitro *tissue growth was enhanced by the addition of IL-17 in a dose-dependent manner.

### IL-17 enhances M-CSF and TNF-α production by ST-derived inflammatory cells in the presence of indomethacin

**Figure 4:**
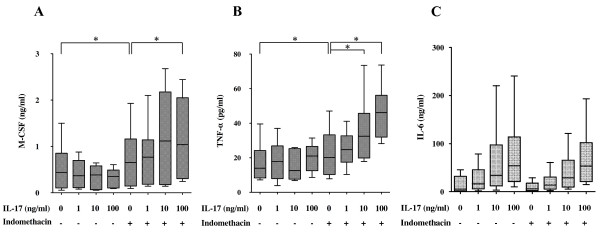
**Effect of interleukin (IL)-17 on the production of macrophage colony-stimulating factor (M-CSF), tumor necrosis factor α (TNF-α) and IL-6**. Synovial tissue (ST)-derived inflammatory cells were incubated with incremental doses of IL-17 in the absence or presence of indomethacin (100 to 1,000 nM) for 1 week. Enzyme-linked immunosorbent assay kits were used to measure the concentration of **(A) **TNF-α, **(B) **M-CSF and IL-6 **(C) **in the culture supernatants derived from seven donors. There were no significant differences in the production of IL-6 between the presence and absence of indomethacin. Box and whisker plots indicate 25th/75th percentile and minimum/maximum, respectively. Bar indicates the median value. *P < 0.05 (by Wilcoxon signed-rank test).

Rheumatoid ST contains a number of proinflammatory cytokines that influence osteoclast formation and bone resorption. Proinflammatory cytokines such as TNF-α and IL-6 stimulate differentiation and activation of osteoclasts, resulting in increased bone resorption. M-CSF is constitutively produced by synovial fibroblasts from RA patients and contributes to the differentiation of synovial macrophages into osteoclasts. We investigated the effect of IL-17 on M-CSF and TNF-α production from ST-derived inflammatory cells. During the cell culture, ST-derived inflammatory cells spontaneously produced M-CSF and TNF-α in the supernatant as described previously [21]. Contrary to our expectation, spontaneous production of both M-CSF and TNF-α was not affected by the addition of IL-17 up to100 ng/ml (Figures 4A and 4B).

As PGE_2 _is known to inhibit the production of M-CSF and TNF-α from macrophages and synovial fibroblasts [22,23], respectively, we examined the effect of IL-17 on the production of M-CSF and TNF-α in the presence of indomethacin to block the effect of endogenous PGE_2_. In the presence of indomethacin, IL-17 significantly enhanced the production of M-CSF and TNF-α in a dose-dependent manner (Figures 4A and 4B), while IL-17-induced IL-6 production was not affected by the addition of indomethacin (Figure 4C).

### IL-17 stimulates osteoclastic bone resorption

**Figure 5:**
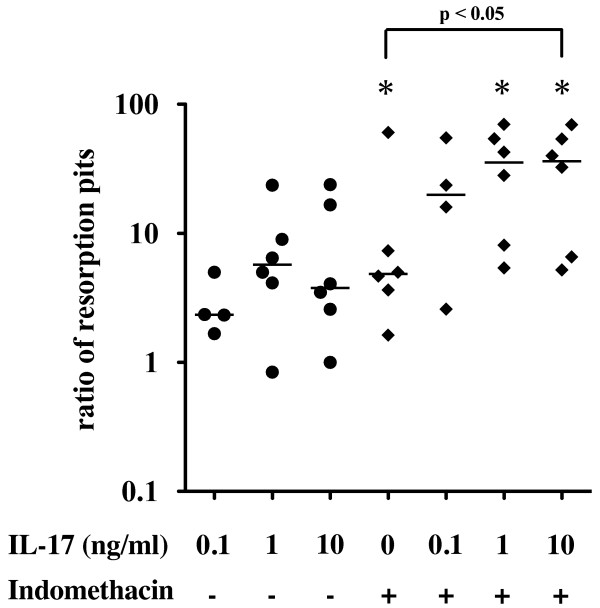
**Effect of interleukin (IL)-17 on the osteoclastogenesis**. Osteoclastic activity was assessed by the development of resorption pits in calcium phosphate-coated slides as described in Materials and methods. Synovial tissue (ST)-derived inflammatory cells (*n *= 6) were cultured with incremental doses of IL-17 in the absence or presence of indomethacin on calcium phosphate-coated slides for 2 weeks and examined for the development of resorption pits. The ratio to the number of resorption pits in medium alone was plotted. Bar indicates the median value. *P < 0.05 vs. medium alone (by Wilcoxon signed-rank test).

We previously showed that ST-derived inflammatory cells in a 1% FCS-containing medium showed spontaneous development of multinucleated giant cells within 2 weeks. They were tartrate-resistant acid phosphatase-positive multinucleated cells and developed numerous resorption pits when incubated on a calcium phosphate-coated slide [21]. Exogenous addition of IL-17 tended to increase the number of resorption pits, but the difference did not reach statistical significance (Figure 5). Indomethacin significantly enhanced the development of resorption pits by the ST-derived inflammatory cells. In the presence of indomethacin, IL-17 significantly increased the number of resorption pits in a dose-dependent manner (Figure 5).

## Discussion

Inflammation in general is fundamentally a protective response against cellular and tissue injury caused by diverse pathological stimuli, and it is closely intertwined with the process of repair. In some circumstances, inflammation and tissue repair are not successfully completed and inflammation perpetuates chronically. RA is characterized by chronic inflammation of the synovial membrane, which results in the development of aggressive granulation tissue, so-called pannus, and the subsequent destruction of cartilage and bone. Pannus tissue is composed mainly of invasive phenotype of FLSs, lymphocytes and activated macrophages, and in the case of bone erosion, monocyte-derived osteoclasts [4]. Cytokine networks and cell-cell interaction, as well as other inflammatory mediators, such as prostanoids, contribute to the development of pannus tissue and osteoclastic activity. This complex system of rheumatoid synovitis includes both positive and negative feedback regulation of inflammatory responses. Therefore, a human cell model that represents this complex system will be useful to study the role of IL-17 in the pathogenesis of RA. We previously established an *ex vivo *cellular model using the ST-derived inflammatory cells, which reproduced pannus-like tissue growth and osteoclastic activity *in vitro*. Using this model, the present study demonstrated that IL-17 enhanced production of proinflammatory cytokines, pannus-like tissue growth and osteoclastic activity by the ST-derived inflammatory cells, while IL-17 simultaneously induced negative feedback regulation through the enhanced production of PGE_2_, a potent deactivator of macrophages and other inflammatory modulator [24]. Inhibition of endogenous PGE_2 _production resulted in the enhancement of pannus growth and osteoclastic activity. Therefore, the net effects of IL-17 may depend upon the balance between the positive and negative regulatory responses.

IL-17 is an important proinflammatory cytokine involved in the pathogenesis of RA. Previous studies have shown that IL-17 is present in rheumatoid synovial fluid and can upregulate several mediators of inflammation, such as TNF-α, IL-1, IL-6, IL-8 and matrix metalloproteinases (MMPs), in FLS. Among other cytokines, both TNF-α and IL-6 have been shown to play a pivotal role in the progression of RA. The importance of TNF-α and IL-6 in the pathogenesis of RA has been established by the clinical experiences with anti-TNF and anti-IL-6 therapy [5-7,11,13]. Blocking TNF-α by either neutralizing mAbs (infliximab and adalimumab) or soluble TNF receptor-immunoglobulin G (IgG)-Fc fusion protein (etanercept) resulted in a rapid and sustained improvement of clinical signs and symptoms in both early and advanced RA. Anti-TNF therapy also prevented radiological progression of joint destruction [8-10]. Anti-IL-6 receptor mAb (tocilizumab) has also been proved to reduce disease activity, even in patients who had insufficient response to anti-TNF therapy [12], and to inhibit the progression of structural joint damage [11,13]. These clinical experiences suggest that there are at least two pathways, TNF-α-dependent and IL-6-dependent, leading to the progression of pannus growth and joint destruction in RA. IL-17 has been shown to stimulate TNF-α and IL-6 expression [16,17], suggesting that IL-17 is an important cytokine located upstream of the two pathways.

PGE_2 _has been established as a regulator of cytokine production by activated macrophages. PGE_2 _inhibits the production of TNF-α, IL-6, IL-8 and IL-12 and downregulates the expression of IL-12 receptor on macrophages [23,25,26]. PGE_2 _downregulates TNF-α and upregulates IL-10 through the EP_2 _and EP_4 _receptors. This effect of PGE_2 _can reverse cytokine disequilibrium from proinflammatory toward anti-inflammatory [24,27]. PGE_2 _has been reported to suppress IL-17-induced TNF-α mRNA expression and protein synthesis in human macrophages and synovial fibroblasts from RA patients via EP_4 _receptor- and EGR-1-mediated inhibition of c-Jun expression [28]. PGE_2 _induces *egr-1 *mRNA expression and protein synthesis by activating transcription factor 2 (ATF-2) dimer via transactivation of the *egr-1 *promoter. IL-17-upregulated promoter activity was largely dependent on ATF-2/c-Jun transactivation. PGE_2 _suppression of IL-17-induced ATF-2/c-Jun transactivation, and DNA binding was dependent on *egr-1*-mediated inhibition of the induced c-Jun expression. While upregulating TNF-α expression, IL-17 also induces cyclooxygenase 2 (COX2)/PGE_2 _expression, which in turn downregulates TNF-α expression. This negative feedback regulation of TNF-α expression by PGE_2 _may be critical in the modulation of the immune and inflammatory responses in RA. The present study has demonstrated that IL-17-induced TNF-α production, pannus-like tissue growth and osteoclastic activity by the ST-derived inflammatory cells were effectively downregulated by the negative feedback loop through PGE_2 _production, while IL-17-induced IL-6 production was not.

PGE_2 _has been shown to inhibit IL-6 production by activated human macrophages [26], while other studies have shown that PGE_2 _enhanced IL-6 production by IL-1β-stimulated human synovial fibroblasts and osteoblasts, as well as chondrocytes [22,29,30]. The present study has shown that the net effect of IL-17 on IL-6 production by the ST-derived inflammatory cells was not affected by the endogenous PGE_2_. This result suggests that the effect of IL-17 is mainly mediated by the IL-6 pathway, while the TNF-α pathway is downregulated by endogenous PGE_2_.

In RA, increased FLS proliferation and/or decreased FLS apoptosis contributes to synovial hyperplasia and pannus growth [31]. IL-17 has been shown to induce proliferation of FLS through the induction of Cyr61, a product of a growth factor-inducible immediate early gene, and the subsequent expression of Bcl-2 that prevents RA FLS apoptosis [32]. COX2-derived PGE_2 _inhibits IL-1/TNF-α-stimulated MMP-1 release from FLSs via inhibition of extracellular signal-regulated kinase (ERK) [33]. On the contrary, COX inhibitors attenuated PGE_2 _inhibition of ERK and enhanced the release of MMP-1 by FLSs [33]. IL-1β and TNF-α stimulate the translocation of p65 and p50 from the cytosol to the nucleus and activate NF-κB in human RA synovial fibroblasts [27]. PGE_2 _inhibits p65 translocation via inhibition of ERK activation and also enhances the expression of IκBα in an ERK-independent manner, suggesting that PGE_2 _inhibits NF-κB activation by both ERK-dependent and ERK-independent mechanisms. These data indicate that PGE_2 _may act to attenuate cytokine-induced inflammatory responses in RA synovial fibroblasts by regulating the localization of specific NF-κB family dimers [27].

In summary, there is accumulating evidence that suggests a molecular cross-talk mechanism involving COX2 and PGE_2 _expression in the resolution of inflammation. Proinflammatory cytokines, including IL-17 and TNF-α, play a critical role in the progression of synovitis and joint destruction, mainly through activation of NF-κB, while they directly induce COX2 and PGE_2 _expression. PGE_2 _upregulates COX2 expression via EP_2 _and EP_4 _receptors and cyclic adenosine monophosphate-dependent signaling pathway, which in turn modulates the production of the proinflammatory molecules. The link between proinflammaory molecules and PGE_2 _could have considerable importance in the regulation of inflammatory cell activation of RA. The paracrine and autocrine feedback mechanisms via COX2, PGE_2_, EP_2 _and EP_4 _could help to avoid the potential pathological damage caused by the excessive production of inflammatory mediators in response to various biological stimuli in RA.

In the present study, we used PGE_1 _instead of PGE_2 _as the exogenous source of cell cultures (Figure 2). Previous studies indicated that PGE_1 _and PGE_2 _are equivalent in terms of biological effects on human synovial fibroblast proliferation [34] and their binding affinity to PGE_2_-specific receptors EP_1_, EP_2_, EP_3 _and EP_4 _[35]. Our preliminary data also shows that both PGE_1 _and PGE_2 _equivalently inhibited both FLS proliferation and *in vitro *pannus-like tissue growth by the ST-derived inflammatory cells in a dose-dependent manner (data not shown). The reason why we have used PGE_1 _instead of PGE_2 _was the fact that we were intending to develop a novel therapeutic strategy utilizing anti-inflammatory effects of PGE_1_. There have been several attempts to use PGE_1 _to treat autoimmune and inflammatory diseases such as adjuvant arthritis [36] and lupus nephritis [37]. We also published the inhibitory effect of lipid microsphere-incorporated PGE_1 _in a collagen-induced arthritis model [38].

Osteoclastic bone resorption is another important feature of pannus tissue in RA. Receptor activator of NF-κB ligand (RANKL) and M-CSF are essential for osteoclastogenesis [39,40]. The expression of RANKL on activated T cells, osteoblasts and synovial fibroblasts contribute to osteoclastic bone resorption in RA patients. M-CSF is constitutively produced by synovial fibroblasts from RA patients and contributes to the differentiation of synovial macrophages into osteoclasts in collaboration with RANKL [41]. In humans, IL-17 induced the expression of both RANK on osteoclast precursors [42] and RANKL on synovial fibroblast [43]. A recent study showed that TNF-induced RANKL expression was IL-6-dependent [44]. On the other hand, both TNF-α and IL-6 also stimulate osteoclastogenesis in a RANKL-independent manner [45,46]. In the present study, we have demonstrated that IL-17 also stimulated M-CSF production by the ST-derived inflammatory cells. The result is consistent with a recent report that IL-17 induced M-CSF expression on human bone marrow-derived mesenchymal stem cells [47].

Another important question is whether IL-17-enhanced osteoclastogenesis under the suppression of endogenous prostanoids is TNF-dependent and/or IL-6-dependent. IL-17 is known to stimulate RANKL expression on fibroblast-like synoviocytes through the induction of IL-6 [44]. On the other hand, IL-17 is reported to induce osteoclast formation through RANK expression on osteoclast precursors [42]. Whether this effect is TNF-dependent and/or IL-6-dependent remains unknown. These questions require further studies including experiments neutralizing TNF-α and IL-6.

Proinflammatory cytokines such as TNF-α and IL-6 have been known to stimulate osteoclastogenesis through enhancing RANKL expression. IL-17, an inducer of TNF-α and IL-6 expression, is also a potent stimulator of osteoclastogenesis in RA. In animal models, it has been reported that TNF-α and IL-1β stimulate osteoclastogenesis through PGE_2 _[48]. Recently, one of these research groups demonstrated that, in contrast to mouse macrophage cultures, PGE_2 _inhibited RANKL-induced human osteoclast formation in CD14^+ ^cell cultures [49]. In our cellular model of RA, we demonstrated that IL-17 enhanced osteoclastogenesis by the ST-derived inflammatory cells only when endogenous prostanoid production was inhibited by indomethacin. The result can be explained by the fact that IL-17-induced TNF-α and M-CSF production was downregulated by the simultaneous induction of endogenous PGE_2_. The present study also leads to a clinically important suggestion that suppression of PGE_2 _by the continuous use of nonsteroidal anti-inflammatory drugs (NSAIDs) such as indomethacin may augment TNF-α pathway-dependent pannus growth and osteoclastic bone resorption, resulting in the joint destruction in RA [24].

## Conclusions

Using a human cellular model of pannus, we have demonstrated that IL-17 induced both proinflammatory cascades, including TNF-α and IL-6, as well as negative feedback regulation by PGE_2 _production. The positive effect of IL-17 on pannus-like tissue growth and osteoclastic activity by the ST-derived inflammatory cells was effectively downregulated by the simultaneously induced endogenous PGE_2_. The negative feedback mechanisms via PGE_2 _could help to avoid the potential pathological damage caused by the excessive production of mediators in response to various biological stimuli such as IL-17 in RA. Whether continuous inhibition of PGE_2 _by the administration of NSAIDs and COX2 inhibitors could augment pannus growth and joint destruction remains to be clarified.

## Abbreviations

COX: cyclooxygenase; FLS: fibroblast-like synoviocyte; IL: interleukin; mAb: monoclonal antibody; M-CSF: macrophage colony-stimulating factor; MMP: matrix metalloproteinase; OPG: osteoprotegrin; PG: prostaglandin; RA: rheumatoid arthritis; RANKL: receptor activator of NF-κB ligand; ST: synovial tissue; Th17: T-helper type 17; TNF: tumor necrosis factor.

## Competing interests

Hidehiro Yamada received research fund from Ono Pharmaceuticals Co. All other authors declare that they have no competing interests.

## Authors' contributions

HI conducted the experimental work, performed the statistical analysis and drafted the manuscript. TNS, HM and SN helped with some experimental work and provided synovial tissues. HY and SO designed and conceived of the study, coordinated the project and drafted the manuscript. All authors read and approved the final manuscript.

## References

[B1] Chu CQ Field M Feldmann M Maini RN Localization of tumor necrosis factor α in synovial tissues and at the cartilage-pannus junction in patients with rheumatoid arthritis Arthritis Rheum 1991 34 1125 1132 10.1002/art.1780340908 1930331

[B2] Buchan G Barrett K Turner M Chantry D Maini RN Feldmann M Interleukin-1 and tumour necrosis factor mRNA expression in rheumatoid arthritis: prolonged production of IL-1α Clin Exp Immunol 1988 73 449 455 3264773 PMC1541753

[B3] Hirano T Matsuda T Turner M Miyasaka N Buchan G Tang B Sato K Shimizu M Maini R Feldmann M Kishimoto T Excessive production of interleukin 6/B cell stimulatory factor-2 in rheumatoid arthritis Eur J Immunol 1988 18 1797 1801 10.1002/eji.1830181122 2462501

[B4] Bromley M Woolley DE Histopathology of the rheumatoid lesion. Identification of cell types at sites of cartilage erosion Arthritis Rheum 1984 27 857 863 10.1002/art.1780270804 6466394

[B5] Maini R St Clair EW Breedveld F Furst D Kalden J Weisman M Smolen J Emery P Harriman G Feldmann M Lipsky P Infliximab (chimeric anti-tumour necrosis factor α monoclonal antibody) versus placebo in rheumatoid arthritis patients receiving concomitant methotrexate: a randomised phase III trial. ATTRACT Study Group Lancet 1999 354 1932 1939 10.1016/S0140-6736(99)05246-0 10622295

[B6] Weinblatt ME Kremer JM Bankhurst AD Bulpitt KJ Fleischmann RM Fox RI Jackson CG Lange M Burge DJ A trial of etanercept, a recombinant tumor necrosis factor receptor:Fc fusion protein, in patients with rheumatoid arthritis receiving methotrexate N Engl J Med 1999 340 253 259 10.1056/NEJM199901283400401 9920948

[B7] Weinblatt ME Keystone EC Furst DE Moreland LW Weisman MH Birbara CA Teoh LA Fischkoff SA Chartash EK Adalimumab, a fully human anti-tumor necrosis factor α monoclonal antibody, for the treatment of rheumatoid arthritis in patients taking concomitant methotrexate: the ARMADA trial Arthritis Rheum 2003 48 35 45 10.1002/art.10697 12528101

[B8] Lipsky PE van der Heijde DM St Clair EW Furst DE Breedveld FC Kalden JR Smolen JS Weisman M Emery P Feldmann M Harriman GR Maini RN Anti-Tumor Necrosis Factor Trial in Rheumatoid Arthritis with Concomitant Therapy Study Group Infliximab and methotrexate in the treatment of rheumatoid arthritis. Anti-Tumor Necrosis Factor Trial in Rheumatoid Arthritis with Concomitant Therapy Study Group N Engl J Med 2000 343 1594 1602 10.1056/NEJM200011303432202 11096166

[B9] Genovese MC Bathon JM Martin RW Fleischmann RM Tesser JR Schiff MH Keystone EC Wasko MC Moreland LW Weaver AL Markenson J Cannon GW Spencer-Green G Finck BK Etanercept versus methotrexate in patients with early rheumatoid arthritis: two-year radiographic and clinical outcomes Arthritis Rheum 2002 46 1443 1450 10.1002/art.10308 12115173

[B10] Breedveld FC Weisman MH Kavanaugh AF Cohen SB Pavelka K van Vollenhoven R Sharp J Perez JL Spencer-Green GT The PREMIER study: a multicenter, randomized, double-blind clinical trial of combination therapy with adalimumab plus methotrexate versus methotrexate alone or adalimumab alone in patients with early, aggressive rheumatoid arthritis who had not had previous methotrexate treatment Arthritis Rheum 2006 54 26 37 10.1002/art.21519 16385520

[B11] Okuda Y Review of tocilizumab in the treatment of rheumatoid arthritis Biologics 2008 2 75 82 19707430 10.2147/btt.s1828 PMC2727785

[B12] Emery P Keystone E Tony HP Cantagrel A van Vollenhoven R Sanchez A Alecock E Lee J Kremer J IL-6 receptor inhibition with tocilizumab improves treatment outcomes in patients with rheumatoid arthritis refractory to anti-tumour necrosis factor biologicals: results from a 24-week multicentre randomised placebo-controlled trial Ann Rheum Dis 2008 67 1516 1523 10.1136/ard.2008.092932 18625622 PMC3811149

[B13] Smolen JS Beaulieu A Rubbert-Roth A Ramos-Remus C Rovensky J Alecock E Woodworth T Alten R Effect of interleukin-6 receptor inhibition with tocilizumab in patients with rheumatoid arthritis (OPTION study): a double-blind, placebo-controlled, randomised trial Lancet 2008 371 987 997 10.1016/S0140-6736(08)60453-5 18358926

[B14] Bush KA Farmer KM Walker JS Kirkham BW Reduction of joint inflammation and bone erosion in rat adjuvant arthritis by treatment with interleukin-17 receptor IgG1 Fc fusion protein Arthritis Rheum 2002 46 802 805 10.1002/art.10173 11920418

[B15] Nakae S Nambu A Sudo K Iwakura Y Suppression of immune induction of collagen-induced arthritis in IL-17-deficient mice J Immunol 2003 171 6173 6177 14634133 10.4049/jimmunol.171.11.6173

[B16] Hwang SY Kim JY Kim KW Park MK Moon Y Kim WU Kim HY IL-17 induces production of IL-6 and IL-8 in rheumatoid arthritis synovial fibroblasts via NF-κB- and PI3-kinase/Akt-dependent pathways Arthritis Res Ther 2004 6 R120 R128 10.1186/ar1038 15059275 PMC400429

[B17] Jovanovic DV Di Battista JA Martel-Pelletier J Jolicoeur FC He Y Zhang M Mineau F Pelletier JP IL-17 stimulates the production and expression of proinflammatory cytokines, IL-β and TNF-α, by human macrophages J Immunol 1998 160 3513 3521 9531313

[B18] Kotake S Udagawa N Takahashi N Matsuzaki K Itoh K Ishiyama S Saito S Inoue K Kamatani N Gillespie MT Martin TJ Suda T IL-17 in synovial fluids from patients with rheumatoid arthritis is a potent stimulator of osteoclastogenesis J Clin Invest 1999 103 1345 1352 10.1172/JCI5703 10225978 PMC408356

[B19] Raza K Falciani F Curnow SJ Ross EJ Lee CY Akbar AN Lord JM Gordon C Buckley CD Salmon M Early rheumatoid arthritis is characterized by a distinct and transient synovial fluid cytokine profile of T cell and stromal cell origin Arthritis Res Ther 2005 7 R784 R795 10.1186/ar1733 15987480 PMC1175027

[B20] Genovese MC Van den Bosch F Roberson SA Bojin S Biagini IM Ryan P Sloan-Lancaster J LY2439821, a humanized anti-interleukin-17 monoclonal antibody, in the treatment of patients with rheumatoid arthritis: a phase I randomized, double-blind, placebo-controlled, proof-of-concept study Arthritis Rheum 2010 62 929 939 10.1002/art.27334 20131262

[B21] Nozaki T Takahashi K Ishii O Endo S Hioki K Mori T Kikukawa T Boumpas DT Ozaki S Yamada H Development of an ex vivo cellular model of rheumatoid arthritis: critical role of CD14-positive monocyte/macrophages in the development of pannus tissue Arthritis Rheum 2007 56 2875 2885 10.1002/art.22849 17763413

[B22] Inoue H Takamori M Shimoyama Y Ishibashi H Yamamoto S Koshihara Y Regulation by PGE_2 _of the production of interleukin-6, macrophage colony stimulating factor, and vascular endothelial growth factor in human synovial fibroblasts Br J Pharmacol 2002 136 287 295 10.1038/sj.bjp.0704705 12010778 PMC1573344

[B23] Di Battista JA Martel-Pelletier J Pelletier J Suppression of tumor necrosis factor (TNF-α) gene expression by prostaglandin E_2_: role Of early growth response protein-1 (Egr-1) Osteoarthritis Cartilage 1999 7 395 398 10.1053/joca.1998.0222 10419778

[B24] Akaogi J Nozaki T Satoh M Yamada H Role of PGE_2 _and EP receptors in the pathogenesis of rheumatoid arthritis and as a novel therapeutic strategy Endocr Metab Immune Disord Drug Targets 2006 6 383 394 17214584 10.2174/187153006779025711

[B25] Takayama K Garcia-Cardena G Sukhova GK Comander J Gimbrone MA Jr Libby P Prostaglandin E_2 _suppresses chemokine production in human macrophages through the EP4 receptor J Biol Chem 2002 277 44147 44154 10.1074/jbc.M204810200 12215436

[B26] Van der Pouw Kraan TC Boeije LC Smeenk RJ Wijdenes J Aarden LA Prostaglandin-E_2 _is a potent inhibitor of human interleukin 12 production J Exp Med 1995 181 775 779 10.1084/jem.181.2.775 7836930 PMC2191857

[B27] Gomez PF Pillinger MH Attur M Marjanovic N Dave M Park J Bingham CO Al-Mussawir H Abramson SB Resolution of inflammation: prostaglandin E_2 _dissociates nuclear trafficking of individual NF-κB subunits (p65, p50) in stimulated rheumatoid synovial fibroblasts J Immunol 2005 175 6924 6930 16272352 10.4049/jimmunol.175.10.6924

[B28] Faour WH Alaaeddine N Mancini A He QW Jovanovic D Di Battista JA Early growth response factor-1 mediates prostaglandin E_2_-dependent transcriptional suppression of cytokine-induced tumor necrosis factor-α gene expression in human macrophages and rheumatoid arthritis-affected synovial fibroblasts J Biol Chem 2005 280 9536 9546 10.1074/jbc.M414067200 15640148

[B29] Takaoka Y Niwa S Nagai H Interleukin-1β induces interleukin-6 production through the production of prostaglandin E_2 _in human osteoblasts, MG-63 cells J Biochem 1999 126 553 558 10467171 10.1093/oxfordjournals.jbchem.a022485

[B30] Wang P Zhu F Konstantopoulos K Prostaglandin E_2 _induces interleukin-6 expression in human chondrocytes via cAMP/protein kinase A- and phosphatidylinositol 3-kinase-dependent NF-κB activation Am J Physiol Cell Physiol 2010 298 C1445 C1456 10.1152/ajpcell.00508.2009 20457835 PMC2889633

[B31] Nakajima T Aono H Hasunuma T Yamamoto K Shirai T Hirohata K Nishioka K Apoptosis and functional Fas antigen in rheumatoid arthritis synoviocytes Arthritis Rheum 1995 38 485 491 10.1002/art.1780380405 7536416

[B32] Zhang Q Wu J Cao Q Xiao L Wang L He D Ouyang G Lin J Shen B Shi Y Zhang Y Li D Li N A critical role of Cyr61 in interleukin-17-dependent proliferation of fibroblast-like synoviocytes in rheumatoid arthritis Arthritis Rheum 2009 60 3602 3612 10.1002/art.24999 19950293

[B33] Pillinger MH Rosenthal PB Tolani SN Apsel B Dinsell V Greenberg J Chan ES Gomez PF Abramson SB Cyclooxygenase-2-derived E prostaglandins down-regulate matrix metalloproteinase-1 expression in fibroblast-like synoviocytes via inhibition of extracellular signal-regulated kinase activation J Immunol 2003 171 6080 6089 14634122 10.4049/jimmunol.171.11.6080

[B34] Clarris BJ Morphological effects of prostaglandins E_1_, E_2 _and F_2_α on fibroblast-like cultures of human synovial cells Experientia 1982 38 350 351 10.1007/BF01949386 6951717

[B35] Kiriyama M Ushikubi F Kobayashi T Hirata M Sugimoto Y Narumiya S Ligand binding specificities of the eight types and subtypes of the mouse prostanoid receptors expressed in Chinese hamster ovary cells Br J Pharmacol 1997 122 217 224 10.1038/sj.bjp.0701367 9313928 PMC1564924

[B36] Zurier RB Quagliata F Effect of prostaglandin E_1 _on adjuvant arthritis Nature 1971 234 304 305 10.1038/234304a0 5003042

[B37] Zurier RB Damjanov I Sayadoff DM Rothfield NF Prostaglandin E_1 _treatment of NZB/NZW F1 hybrid mice. II. Prevention of glomerulonephritis Arthritis Rheum 1977 20 1449 1456 10.1002/art.1780200802 144506

[B38] Moriuchi-Murakami E Yamada H Ishii O Kikukawa T Igarashi R Treatment of established collagen induced arthritis with prostaglandin E_1 _incorporated in lipid microspheres J Rheumatol 2000 27 2389 2396 11036835

[B39] Udagawa N Kotake S Kamatani N Takahashi N Suda T The molecular mechanism of osteoclastogenesis in rheumatoid arthritis Arthritis Res 2002 4 281 289 10.1186/ar431 12223101 PMC128939

[B40] Quinn JM Elliott J Gillespie MT Martin TJ A combination of osteoclast differentiation factor and macrophage-colony stimulating factor is sufficient for both human and mouse osteoclast formation in vitro Endocrinology 1998 139 4424 4427 10.1210/en.139.10.4424 9751528

[B41] Danks L Sabokbar A Gundle R Athanasou NA Synovial macrophage-osteoclast differentiation in inflammatory arthritis Ann Rheum Dis 2002 61 916 921 10.1136/ard.61.10.916 12228163 PMC1753924

[B42] Adamopoulos IE Chao CC Geissler R Laface D Blumenschein W Iwakura Y McClanahan T Bowman EP Interleukin-17A upregulates receptor activator of NF-κB on osteoclast precursors Arthritis Res Ther 2010 12 R29 10.1186/ar3238 20167120 PMC2875663

[B43] Tunyogi-Csapo M Kis-Toth K Radacs M Farkas B Jacobs JJ Finnegan A Mikecz K Glant TT Cytokine-controlled RANKL and osteoprotegerin expression by human and mouse synovial fibroblasts: fibroblast-mediated pathologic bone resorption Arthritis Rheum 2008 58 2397 2408 10.1002/art.23653 18668542

[B44] Hashizume M Hayakawa N Mihara M IL-6 trans-signalling directly induces RANKL on fibroblast-like synovial cells and is involved in RANKL induction by TNF-α and IL-17 Rheumatology (Oxford) 2008 47 1635 1640 10.1093/rheumatology/ken363 18786965

[B45] Kudo O Fujikawa Y Itonaga I Sabokbar A Torisu T Athanasou NA Proinflammatory cytokine (TNFα/IL-1α) induction of human osteoclast formation J Pathol 2002 198 220 227 10.1002/path.1190 12237882

[B46] Kudo O Sabokbar A Pocock A Itonaga I Fujikawa Y Athanasou NA Interleukin-6 and interleukin-11 support human osteoclast formation by a RANKL-independent mechanism Bone 2003 32 1 7 10.1016/S8756-3282(02)00915-8 12584029

[B47] Huang H Kim HJ Chang EJ Lee ZH Hwang SJ Kim HM Lee Y Kim HH IL-17 stimulates the proliferation and differentiation of human mesenchymal stem cells: implications for bone remodeling Cell Death Differ 2009 16 1332 1343 10.1038/cdd.2009.74 19543237

[B48] Sakuma Y Tanaka K Suda M Yasoda A Natsui K Tanaka I Ushikubi F Narumiya S Segi E Sugimoto Y Ichikawa A Nakao K Crucial involvement of the EP4 subtype of prostaglandin E receptor in osteoclast formation by proinflammatory cytokines and lipopolysaccharide J Bone Miner Res 2000 15 218 227 10.1359/jbmr.2000.15.2.218 10703923

[B49] Take I Kobayashi Y Yamamoto Y Tsuboi H Ochi T Uematsu S Okafuji N Kurihara S Udagawa N Takahashi N Prostaglandin E_2 _strongly inhibits human osteoclast formation Endocrinology 2005 146 5204 5214 10.1210/en.2005-0451 16150915

